# In situ controlled rapid growth of novel high activity TiB_2_/(TiB_2_–TiN) hierarchical/heterostructured nanocomposites

**DOI:** 10.3762/bjnano.8.211

**Published:** 2017-10-10

**Authors:** Jilin Wang, Hejie Liao, Yuchun Ji, Fei Long, Yunle Gu, Zhengguang Zou, Weimin Wang, Zhengyi Fu

**Affiliations:** 1 School of Materials Science and Engineering, Key Laboratory of Nonferrous Materials and New Processing Technology of Ministry of Education, Guilin University of Technology, Guilin 541004, China; 2 Nano and Ceramic Materials Research Center, Wuhan Institute of Technology, Wuhan 430073, China,; 3 The State Key Laboratory of Advanced Technology for Materials Synthesis and Processing, Wuhan University of Technology, Wuhan 430070, China

**Keywords:** chemical activity, hierarchical/heterostructures, self-propagating high-temperature synthesis, TiB_2_, TiN

## Abstract

In this work, a reaction coupling self-propagating high-temperature synthesis (RC-SHS) method was developed for the in situ controlled synthesis of novel, high activity TiB_2_/(TiB_2_–TiN) hierarchical/heterostructured nanocomposites using TiO_2_, Mg, B_2_O_3_, KBH_4_ and NH_4_NO_3_ as raw materials. The as-synthesized samples were characterized using X-ray diffraction (XRD), scanning electron microscope (SEM), X-ray energy dispersive spectroscopy (EDX), transition electron microscopy (TEM), high-resolution TEM (HRTEM) and selected-area electron diffraction (SAED). The obtained TiB_2_/TiN hierarchical/heterostructured nanocomposites demonstrated an average particle size of 100–500 nm, and every particle surface was covered by many multibranched, tapered nanorods with diameters in the range of 10–40 nm and lengths of 50–200 nm. In addition, the tapered nanorod presents a rough surface with abundant exposed atoms. The internal and external components of the nanorods were TiB_2_ and TiN, respectively. Additionally, a thermogravimetric and differential scanning calorimetry analyzer (TG-DSC) comparison analysis indicated that the as-synthesized samples presented better chemical activity than that of commercial TiB_2_ powders. Finally, the possible chemical reactions as well as the proposed growth mechanism of the TiB_2_/(TiB_2_–TiN) hierarchical/heterostructured nanocomposites were further discussed.

## Introduction

Refractory materials such as borides, nitrides and carbides have attracted great attention for advanced engineering applications due to their exceptional hardness, thermal and chemical stability at high temperatures [1–3]. For example, titanium diboride (TiB_2_) processes high hardness and a high melting point, good chemical and metallurgical stability, as well as excellent electrical and thermal conductivity [4–6]. On the other hand, titanium nitride (TiN) has some attractive properties, such as high hardness, low electrical resistivity, excellent wear and corrosion resistance [1–2,7]. Therefore, it is expected that these unique properties will make TiB_2_/TiN composites an attractive prospect for practical applications in many fields such as super-hard materials, electrodes, wear resistance materials, armor plates, jet engine parts and high temperature ceramic components [1,3,7–8].

The previous researches were primarily focused on the preparation and performance of TiB_2_/TiN composite ceramics or films. However, there are few reports about the preparation of the raw powders of TiB_2_/TiN composites. It is common knowledge that high activity powders are the indispensable prerequisite for the preparation of the corresponding composites with distinguished properties [9–10]. Thus, in our opinion, the understanding of the fabrication of homogeneously dispersed, high activity TiB_2_/TiN nanocomposite powders is of great importance.

As for the preparation of TiB_2_/TiN composite powders, the common synthesis routes are combustion synthesis methods and high-energy ball milling assisted methods with heat treatment using Ti, B, BN and TiH_2_ as raw materials [1–3,11–13]. Based on the previous works, it is known that the high-energy ball milling assisted heat treatment method requires long milling time (30–40 h) and high energy consumption. In addition, although the combustion synthesis method is suitable for industrialized production of inorganic ceramic powders, the raw materials used in this method are expensive. Even more remarkable, the TiB_2_/TiN composite powders synthesized by these two above-mentioned preparation methods also display conventional particle morphology.

Recently, several kinds of inorganic nanomaterials have been prepared in our research group using a developed reaction coupling self-propagating high-temperature synthesis (RC-SHS) technology [9–10,14]. The results showed that the heat of the reaction intensity, morphology, purity, particle size and dispersibility of the samples could be controlled through changing the coupling reaction (endothermic reaction) ratio. Given the current problems regarding the preparation of TiB_2_/TiN composite powders, based on our previous research idea, the production of distinctive TiB_2_/TiN products synthesized via this RC-SHS method are desired.

In this paper, we propose an effective method for the in situ controlled, rapid synthesis of novel TiB_2_/(TiB_2_–TiN) hierarchical/heterostructured nanocomposites via the RC-SHS method. The phase, element composition, morphology, microstructure and chemical activity of the as-synthesized products were investigated in detail by various characterization methods. In addition, further comparative experiments were carried out to study the relation between the endothermic reaction rate and the morphology/microstructure/composition of the samples. Moreover, based on the obtained experimental results and previous work, the possible chemical reactions as well as the appropriate growth mechanism of the TiB_2_/(TiB_2_–TiN) hierarchical/heterostructured nanocomposites were also discussed.

## Results and Discussion

**Figure 1:**
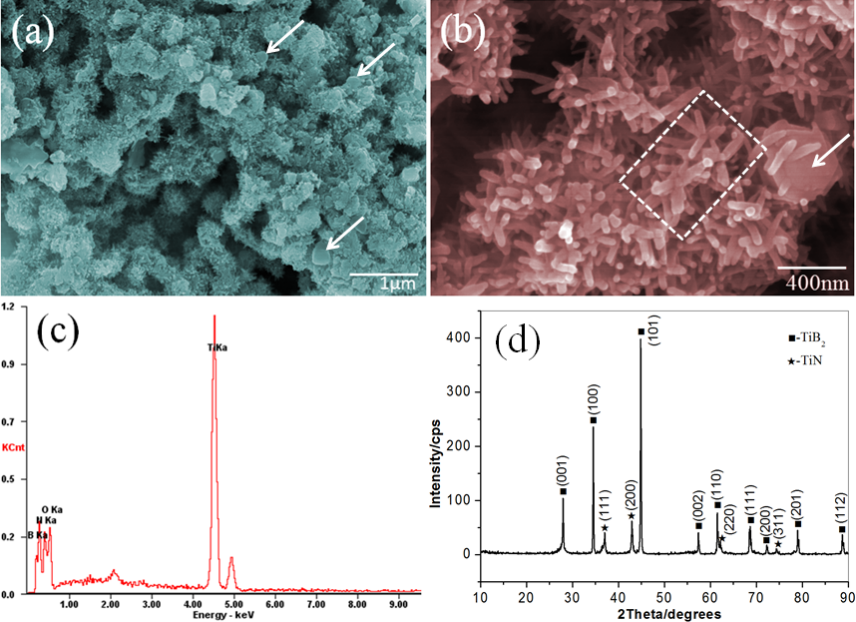
Typical field emission scanning electron microscope (FSEM) images (a, b), EDX spectra (c) and XRD pattern (d) of the as-synthesized TiB_2_/(TiB_2_–TiN) samples. Scale bars: (a) 1 μm and (b) 400 nm.

Figure 1a,b gives the typical field emission scanning electron microscope (FSEM) images of the as-prepared TiB_2_/(TiB_2_–TiN) samples where the endothermic rate is 40%. It is obvious that the samples demonstrated interesting hierarchical structures which were composed of grains and short rods. In detail, these grains revealed particle sizes of about 100–500 nm. The surface of the grain was covered by many tapered nanorods with a diameter of 10–40 nm and a length of 50–100 nm (indicated by arrows). In addition, the grain that was originally located at the center of the hierarchical structure eventually disappeared completely and transformed into a scattering multibranched structure (marked with frame). Moreover, hierarchical structures were well-distributed without agglomeration. The corresponding X-ray energy dispersive spectroscopy (EDX) spectrum (Figure 1c) reveals significant elemental B, Ti, N and O. The existence of O could be attributed to the surface hydrolysis and oxidation of the hierarchical structures. Figure 1d displays the typical X-ray diffraction (XRD) pattern of the as-prepared TiB_2_/(TiB_2_–TiN) samples where the endothermic rate is 40%. Nine peaks could be assigned to characteristic planes of TiB_2_ (JCPDF card No.65-1073) [15]. The other four peaks could be indexed to corresponding planes of TiN (JCPDF card No.65-5774) [16]. Additionally, the peaks of other byproducts, such as MgO, were not detected in this pattern.

**Figure 2:**
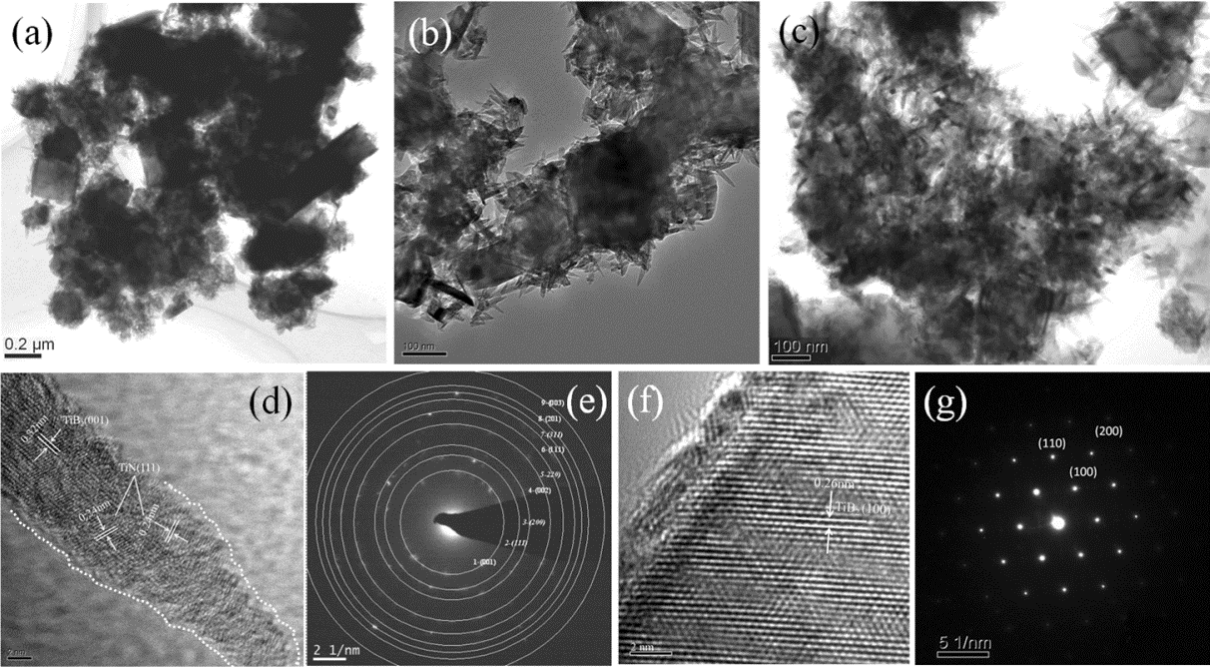
Typical TEM (a–c), and high-resolution TEM (d, f) images and selected-area electron diffraction (SAED) patterns (e, g) of the as-synthesized TiB_2_/(TiB_2_–TiN) samples. Scale bars: (a) 0.2 μm, (b) 100 nm, (c) 100 nm, (d) 2 nm and (f) 2 nm.

Figure 2a–c presents typical transition electron microscopy (TEM) images of the as-prepared TiB_2_/(TiB_2_–TiN) samples where the endothermic rate is 40%. Figure 2a shows a low magnification image, and Figure 2b,c show high-magnification images. The sizes of solid grains were in the range of 100–200 nm. The nanorods also displayed solid structures with a length of 50–150 nm and tapering diameters from 5 nm to 30 nm.

In order to further determine the internal microstructure and phase constitution of the as-synthesized samples, the high-resolution transition electron microscopy (HRTEM) (Figure 2d,f) and selected-area electron diffraction (SAED) (Figure 2e,g) analysis of the tapered nanorods and grains was performed, respectively. As shown in Figure 2d, the interlayer distances of the single tapered nanorod were measured as *d*_1_ = 0.319 nm and *d*_2_ = 0.241 nm, corresponding to the (001) plane of TiB_2_ and the (111) plane of TiN, respectively. The corresponding SAED pattern (Figure 2e) indicated that the five diffraction rings numbered 1, 4, 6, 8 and 9 were assigned to (001), (002), (111), (201) and (003) lattice planes of TiB_2_ (PDF card No.65-1073) [15]. The other four diffraction rings numbered 2, 3, 5 and 7 were attributed to (111), (200), (220) and (311) lattice planes of TiN (PDF card No.65-5774) [16].

It was worth noting that the tapered nanorods present a rough surface with abundant exposed atoms (Figure 2d), which was attributed to the different growth rates of the neighboring atom layers along the direction parallel to the crystal plane (pointed out by dash lines) [17]. It is possible that these exposed atoms could serve as high activity positions and might be used in future research in the fields of proton exchange membrane fuel cells, surface chemical modification and functionalization [18–19]. Moreover, the internal and external components were TiB_2_ and TiN, respectively. The crystal planes of TiB_2_ were found along the length direction of the nanorods and formed an angle with that of TiN.

As for the grains, the typical HRTEM image (Figure 2f) presented clear lattice fringes with different interplanar spacings of about 0.26 nm, which was close to that of the (100) plane of TiB_2_. The corresponding SAED pattern (Figure 2g) indicated that the sample was a TiB_2_ single crystal with high crystallinity. In general, the HRTEM and SAED analysis results of the hierarchical/heterostructured nanocomposites corresponded with those of the above-mentioned XRD, EDX and FSEM characterizations.

### Effects of endothermic rate

**Figure 3:**
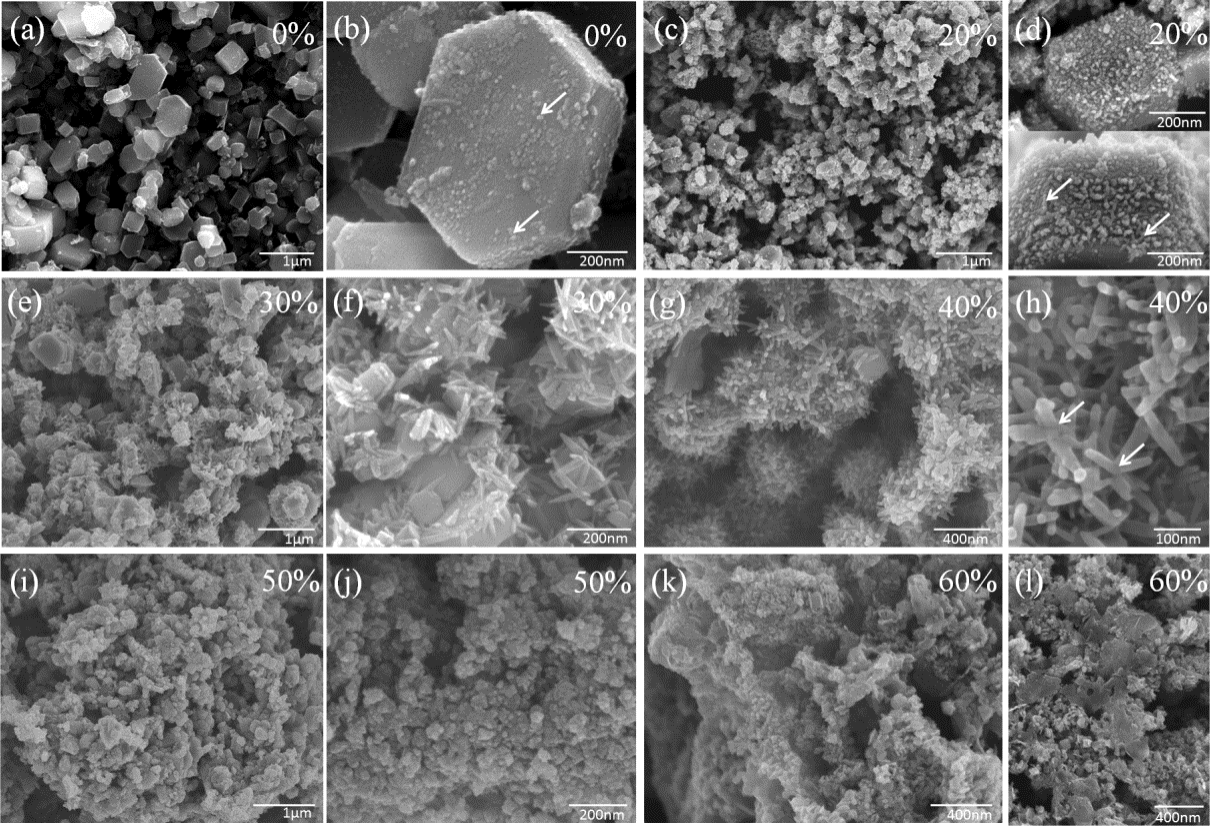
Typical FSEM images of the as-synthesized TiB_2_/(TiB_2_–TiN) samples with different endothermic rates (a,b) 0%; (c,d) 20%; (e,f) 30%; (g, h) 40%; (h,i) 50%; (j,k) 60%. Scale bars: (a) 1 μm, (b) 200 nm, (c) 1 μm, (d) 200 nm, (e) 1 μm, (f) 200 nm, (g) 400 nm, (h) 100 nm, (i) 1 μm, (j) 200 nm, (k) 400 nm and (l) 400 nm.

Figure 3 shows typical FSEM images of the as-prepared TiB_2_–TiN samples with different endothermic rates. When the endothermic rate was 0%, the samples presented a clear typical hexagonal prism morphology of well-crystallized TiB_2_ with a broad particle size of 0.02–1.5 μm (Figure 3a,b). It is worth noting that some nanoparticles (about 5 nm in diameter) existed on the surface of the hexagonal prism (pointed out by arrows in Figure 3b). When the endothermic rate reached 20%, the hexagonal prisms demonstrated slight agglomeration with the smaller particle size ranging from 200–600 nm (Figure 3c,d). Although the contour of the hexagonal prism could also be observed, the surface of the hexagonal prism became much rougher, which was attributed to the large number of nanoparticles. In addition, it was interesting that the some nanoparticles began to self-assemble into short rods (pointed out by arrows in Figure 3d). With the increase of the endothermic rate (30%), many short rods emerged on the surface of the hexagonal prisms (Figure 3e,f). With the growth of short rods, some hexagonal prisms became smaller or were completely covered with short rods. When the endothermic rate was 40%, the novel hierarchical/heterostructures emerged, which were composed of hexagonal prism grains and short rods (Figure 3g,h). Additionally, the grains that originally existed at the center of the hierarchical structures eventually disappeared completely and transformed into scattering multibranched structures (marked with arrows in Figure 3h); however, when the endothermic rate was increased to 50%, the hexagonal prisms and short rods both vanished, and only irregularly shaped particles with poor crystallinity were found (Figure 3i,j). Moreover, the higher endothermic rate (60%) led to various morphologies of samples (Figure 3k,l). It is likely that the reaction was incomplete, which then caused the generation of impurities and/or byproducts [9–10].

Given the above-mentioned comparative experimental analysis results, it could be shown that the morphology, particle size, microstructure and purity of TiB_2_/(TiB_2_–TiN) samples could be effectively controlled through the in situ reaction coupling self-propagating high-temperature synthesis (RC-SHS) method. The results were found to be in agreement with that of our previous studies [9–10,14].

### Chemical reactions and growth mechanism

During the growth process of the TiB_2_/(TiB_2_–TiN) hierarchical/heterostructured nanocomposites, the following chemical reactions are applied:

[1]
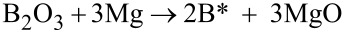


[2]
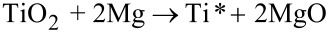


[3]
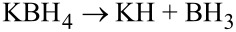


[4]
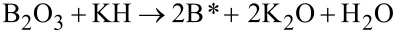


[5]
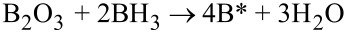


[6]
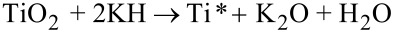


[7]
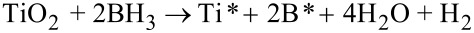


[8]
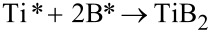


[9]
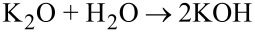


[10]
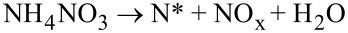


[11]
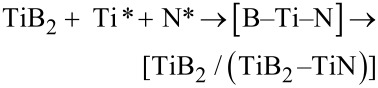


At the beginning of the RC-SHS process, the exothermic magnesium reaction occurs and produces chemically active B* and Ti* with a large amount of heat (Equation 1 and Equation 2) [14,20]. The heat leads to the decomposition of KBH_4_ and generates KH/BH_3_ with strong reduction capacity (Equation 3) [21–23]. The KH/BH_3_ reacts with the residual B_2_O_3_ and TiO_2_, then forms B*, Ti*, K_2_O, H_2_O and H_2_ (Equation 4–7). The B* combines with Ti* to produce TiB_2_ crystals (Equation 8). KOH is also produced by the reaction between K_2_O and H_2_O (Equation 9) [13]. At the same time, the high temperature could promote NH_4_NO_3_ splitting into active N*, NO*_x_* and H_2_O (Equation 10) [24–25]. Under the special reaction coupling self-propagating high-temperature environment, N* and the rest of Ti* could be deposited on the surface of the TiB_2_ crystals, and finally, the novel TiB_2_/(TiB_2_–TiN) hierarchical/heterostructured nanocomposites are the product (Equation 11).

**Figure 4:**
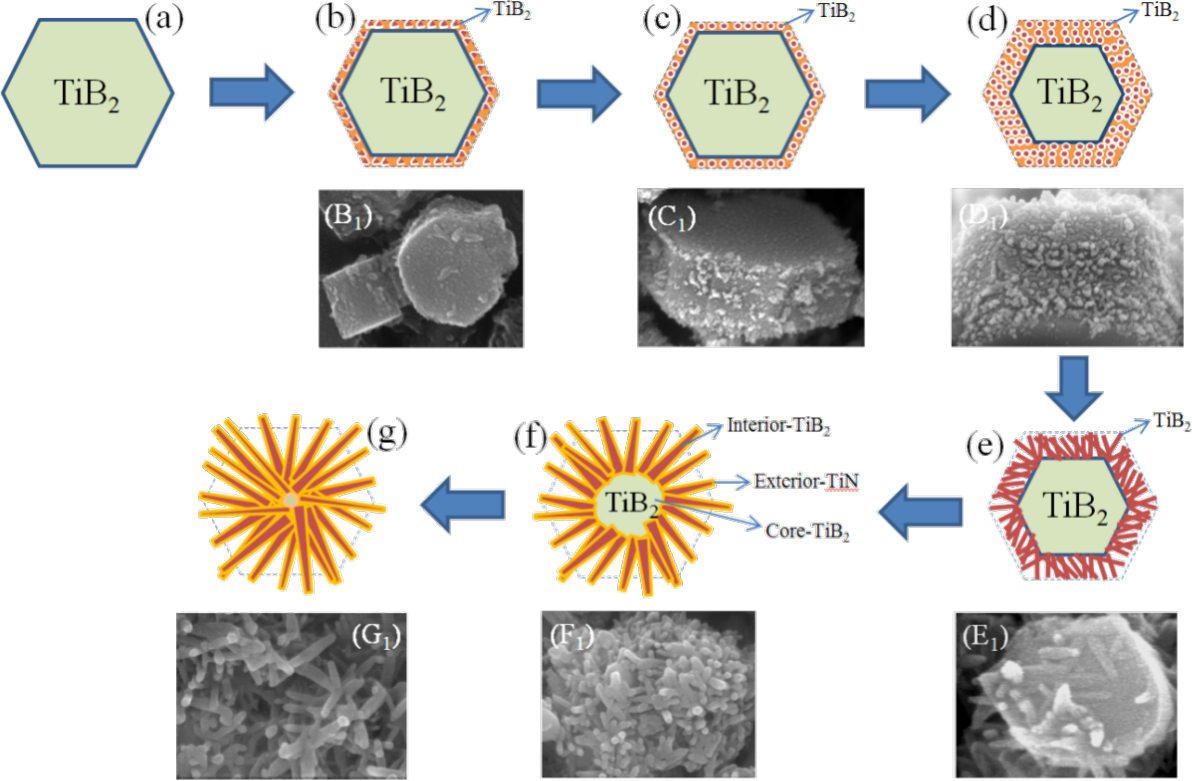
Proposed schematic illustration and the corresponding FSEM images related to the formation process of the as-synthesized TiB_2_/(TiB_2_–TiN) samples. (a) TiB_2_ hexagonal prism crystal. (b) (B_1_) The surface layer was broken into fragments. (c) (C_1_) TiB_2_ fragments melted into small droplets. (d) (D_1_) Small droplets self-assembled into bubble-like short chains. (e) (E_1_) Bubble-like short chains transformed into nanorods. (f) (F_1_) TiN formed on the surface on the nanorods. (g) (G_1_) TiB_2_/(TiB_2_–TiN) hierarchical/heterostructured nanocomposites are the resulting product.

The basic principle of the self-propagating high-temperature synthesis technology has been studied for many years and has achieved some meaningful success [26–27]. However, the corresponding deeper research regarding the growth mechanism of the desired inorganic materials with hierarchical and/or heterostructures has been rarely reported. In this work, first, high quality TiB_2_ hexagonal prism crystals were prepared according to the above mentioned reaction coupling self-propagating high-temperature synthesis principle (Figure 4a). The SHS reaction system creates a special environment where the surface layer of the formed TiB_2_ crystals can be broken into fragments due to the infiltration and erosion of the high concentration of N*, Ti*and H_2_ vapors (Figure 4b) [28–30]. Under high temperature, the TiB_2_ fragments melted into many small droplets (Figure 4c). And the energy balance at the surface of the TiB_2_ crystal depends strongly on the changes in the external environment conditions. Thus, the small droplets self-assembled into bubble-like short chains (Figure 4d) followed by rods (Figure 4e) in order to satisfy the minimized surface energy [31]. Then the N* and the rest of the Ti* could be co-precipitated on the surface of the TiB_2_ short rods to generate the TiN outer layers (Figure 4f,g). The different atom deposition rates of the neighboring atom layers along the direction parallel to the crystal plane results in nanorods with a rough surface with abundant exposed atoms (Figure 2d) [17]. In consideration of the remarkably short time of the SHS processes, the Ti, N and B atoms would likely not have enough time to diffuse and rearrange. Consequently, the crystallinity becomes gradually worse from the outer to the inside of the TiB_2_/TiN rod heterostructure (Figure 2d).

**Figure 5:**
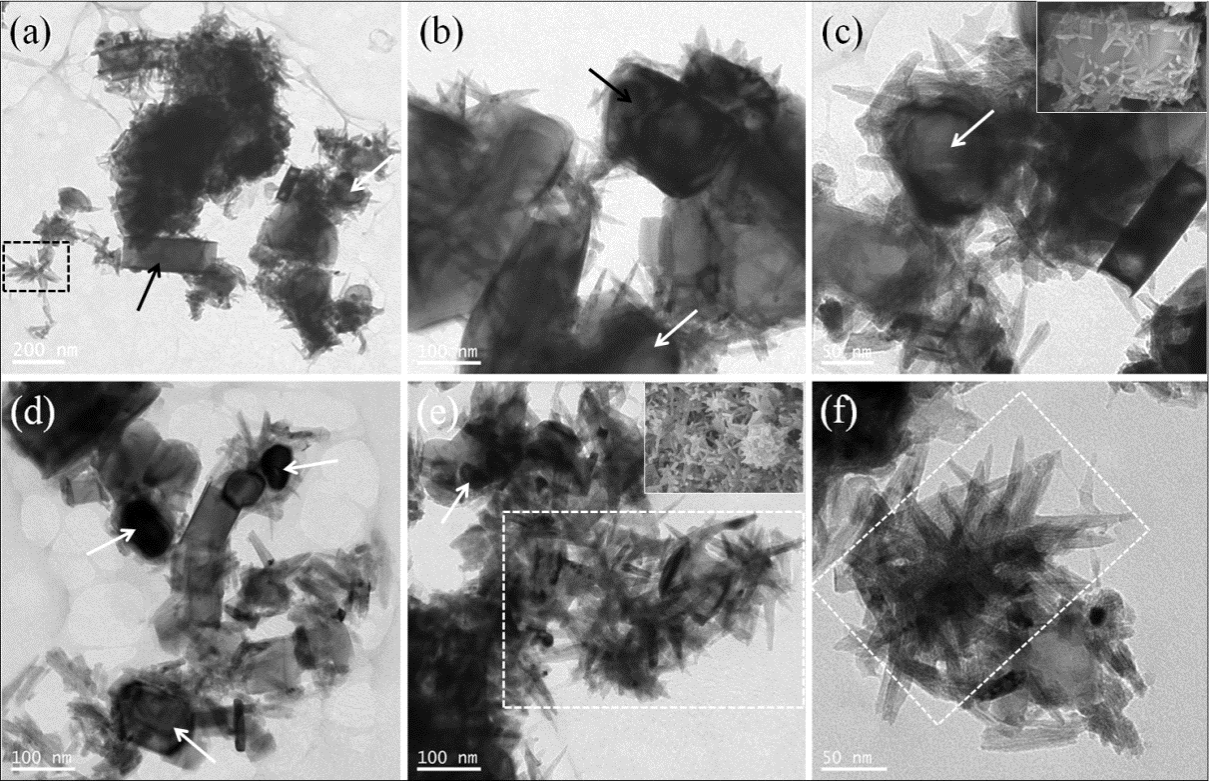
Typical TEM and FSEM images of the as-synthesized TiB_2_/(TiB_2_–TiN) samples with different thickness of the infiltration and erosion layers. Scale bars: (a) 200 nm, (b) 100 nm, (c) 50 nm, (d) 100 nm, (e) 100 nm and (f) 50 nm.

Moreover, it was obvious that the grains that were previously at the center of the as-synthesized TiB_2_/(TiB_2_–TiN) hierarchical/heterostructured nanocomposites displayed different particle sizes (Figure 5, indicated with arrows); however, the above-mentioned growth mechanism was still not be able to explain the phenomenon. In fact, the particle size of the grain depended on the size of the initially formed TiB_2_ crystal. According to this peculiar small size effect [32–34], the small particle has a higher specific surface area and chemical activity than that of the large particle. Therefore, under the same conditions, the relative thickness of the infiltration and erosion layer was different between large and small particles. The smaller the TiB_2_ crystals, the thicker the infiltration and erosion layers should be (Figure 5, indicated with arrows). In other words, the quantity of residual TiB_2_ grain is less when the initial TiB_2_ particle is small (Figure 4f). When the initially formed TiB_2_ was infiltrated and eroded completely, the TiB_2_ grain at the core of the hierarchical/heterostructured nanocomposites disappeared (Figure 4g), and finally, only scattering multibranched structures sharing one head were obtained (Figure 5a,e,f, marked with dashed frames).

### Chemical activity

**Figure 6:**
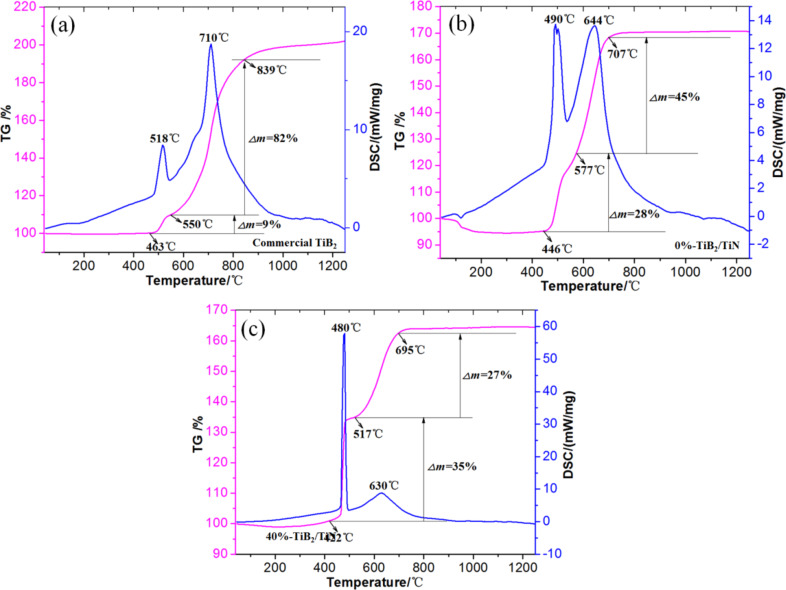
TG-DSC results of three representative samples (a-commercial high purity TiB_2_ powders, b-TiB_2_/(TiB_2_–TiN) powders of 0% endothermic rate, c-TiB_2_/(TiB_2_–TiN) powders of 40% endothermic rate).

In order to study the chemical activity of the as-synthesized TiB_2_/(TiB_2_–TiN) hierarchical/heterostructured nanocomposites, thermogravimetric and differential scanning calorimetry analyzer (TG-DSC) thermal analysis comparison experiments have been employed in flowing air. Figure 6a–c shows the TG-DSC results of three representative samples (#1 commercial high purity TiB_2_ powders with an average particle size of 5 μm, #2 TiB_2_/(TiB_2_–TiN) powders of 0% endothermic rate with an average particle size of 0.9 μm, #3 TiB_2_/(TiB_2_–TiN) powders of 40% endothermic rate with an average particle size of 0.15 μm). Table 1 also summarizes the major related TG-DSC results and the average particle sizes of the samples (see Supporting Information File 1). The oxidation curves of the samples were typical of those reported in previous literature [35–38]. The oxidation process can be divided into three stages. Firstly, TiB_2_ is oxidized to TiO_2_ and solid B_2_O_3_ [36,39]. Secondly, when the temperature reaches the melting point, the B_2_O_3_ begins to transform into liquid [37]. The molten liquid B_2_O_3_ acts as a protective layer and restrains the reaction between remaining TiB_2_ particle and O_2_ [36,40]. Thirdly, with the increase of temperature, the evaporation of liquid B_2_O_3_ becomes more extensive due to its higher vapor pressure [36–37]. However, there were also some significant differences in the TG and DSC results. The oxidation of samples #1, #2 and #3 started at 463 °C, 446 °C and 422 °C (TG plots), which correspond to the exothermic peaks of about 518 °C (peak #1), 490 °C (peak #2) and 480 °C (peak #3), respectively (DSC curves). In addition, the other weight gain temperature ranges of the three samples (#1 550–839 °C, #2 577–707 °C, #3 517–695 °C) in the TG plots were assigned as exothermic reactions at 710 °C, 644 °C and 630 °C in the DSC analysis. Besides, the proportion of the released heat for peak 1 increased in turn from sample #1, #2 to #3. The same trends were evident with the weight change from the TG analysis.

Based on the TG-DSC analysis results and the previous research, sample #3 could most easily be oxidized and revealed the highest oxidation degree, but the opposite was true for sample #1. This implies that the chemical activity sequence of the three samples was #1 < #2 < #3. In our opinion, there were three reasons that account for this phenomenon: (1) according to the peculiar small size effect, the smaller particles demonstrate higher specific surface areas than those of larger particle [32–34]; (2) the infiltration and erosion of N*, Ti* and H_2_ vapors [28–30] in the RC-SHS process could lead to the rough metastable surface with abundant active atoms and/or defects; or (3) the novel hierarchical/heterostructured nanocomposites with a uniform particle size distribution may be favorable for improving the chemical activity of samples.

## Conclusion

Novel TiB_2_/(TiB_2_–TiN) hierarchical/heterostructured nanocomposites were fabricated via a special reaction coupling self-propagating high temperature synthesis (RC-SHS) method where in situ control of the material properties was established. The as-synthesized samples consisted of TiB_2_ nanograins as the core/seed and TiB_2_/(TiB_2_–TiN) composite nanorods which covered this core. The TiB_2_/(TiB_2_–TiN) composite nanorods also presented a rough surface with abundant exposed active atoms and/or defects. Additionally, based on the obtained experimental results and the previous studies, the proposed chemical reaction processes and the corresponding growth model of the hierarchical/heterostructured nanocomposites were justified. Moreover, the high chemical activity of the samples was also confirmed by TG-DSC analysis. This special highly active material could possibly be further used in the research fields of proton exchange membrane fuel cells, structural and functional performance integrated composite ceramics and/or films, surface chemical modification and functionalization.

Another point worth noting is that the detailed comparison experiments have demonstrated that the reaction coupling self-propagating high temperature synthesis technology could effectively be used to control the morphology, particle size, dispersibility and purity of the as-synthesized samples. Therefore, this work not only provides new experimental and theoretical evidence for the scientific rationality of this new technology, but also further expands the variety of materials available from single inorganic nanocrystals to binary hierarchical/heterostructured nanocomposites.

## Experimental

### Reaction coupling principles and the theoretical calculations

The reaction coupling principles have been reported in our previous work [9–10,14]. Generally, it is essential to find two kinds of reactions: one reaction is exothermic, while the other is endothermic. The heat energy of the whole RC-SHS system can be controlled by changing the endothermic reaction ratio. The endothermic rate is the percentage of the resulting material (TiB_2_) prepared through an endothermic reaction with respect to the total desired material (TiB_2_). In this work, the two kinds of reactions could be designed as follows: Equation 12 is associated with a traditional magnesium exothermic reaction, while Equation 13 is an endothermic reaction. The standard enthalpies of Equation 12 and Equation 13 are calculated as −15.88 kJ/(g TiB_2_) and 5.89 kJ/(g TiB_2_), respectively. Besides, ammonium nitrate and nitrogen are applied as the nitrogen source. Finally, the whole reaction of B_2_O_3_/TiO_2_/Mg/KBH_4_/NH_4_NO_3_/N_2_ can be described as follows in Equation 14.

[12]
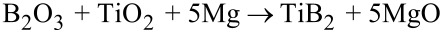


[13]
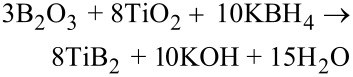


[14]
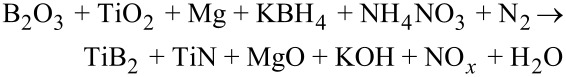


**Figure 7:**
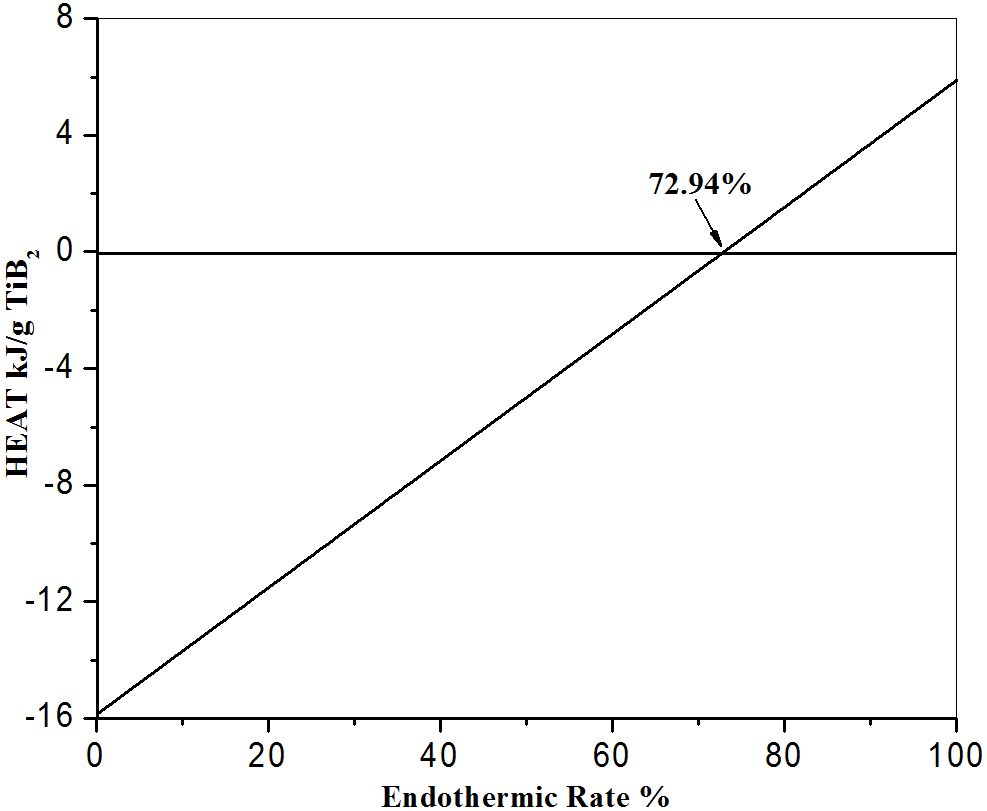
Detailed relationship between the endothermic rate and the total heat effect.

Figure 7 presents the relationship between the endothermic rate and thermal effect. When the endothermic rate reaches 72.94%, the heat effect of the whole reaction system achieves equilibrium.

### Synthesis techniques

The raw materials TiO_2_, Mg, B_2_O_3_, KBH_4_ and NH_4_NO_3_ were of analytical grade and purchased from Sinopharm Chemical Reagent Co., Ltd., China without further treatment. In a typical procedure, TiO_2_, Mg and B_2_O_3_ were milled for 2 h by a planet-type ball mill (the weight rate between stainless steel ball and mixture powders was 30:1, rotating rate was 250 rpm). The obtained milled products were mixed with KBH_4_ and NH_4_NO_3_. Then the mixture (mole ratio B/Ti/N was 2.65:1.34:0.7) was pressed into a cylinder with a diameter of 30 mm. The cylinder was heated in a nitrogen atmosphere at 700–850 °C for 8–15 min in a self-designed SHS furnace. After cooling to room temperature naturally, the crude product was collected and washed with hydrochloric acid, ethanol and distilled water. Finally, TiB_2_/(TiB_2_–TiN) nanocomposite powders were collected by drying in vacuum at 80 °C for 24 h.

### Characterization

The phase structure of the samples was characterized using powder X-ray diffraction (XRD) with Cu Kα radiation. The morphology and microstructure of the samples were investigated using a Hitachi S4800 field emission scanning electron microscope (FSEM), a Philips CM12 transmission electron microscope (TEM), a JEOL JEM-2100F high-resolution transmission electron microscope (HRTEM) and the selected-area electron diffraction (SAED) instrument attached on the Philips CM12 TEM. The elemental composition and content of the samples was analyzed using an X-ray energy dispersive spectroscopy (EDX) attached to the S4800 FSEM. The chemical activity of the samples was measured with the help of the Netzsch STA 449F3 thermogravimetric and differential scanning calorimetry analyzer (TG-DSC) at a heating rate of 10 °C/min.

## Supporting Information

File 1 Additional TG-DSC results.
