# Supplementary material for: In situ controlled rapid growth of novel high activity TiB2/(TiB2–TiN) hierarchical/heterostructured nanocomposites
Source: Beilstein J Nanotechnol. 2017 Oct 10;8:2116–25. doi: 10.3762/bjnano.8.211 (PMC5647741; doi:10.3762/bjnano.8.211)
Supplement: File 1 — Additional TG-DSC results. [file Beilstein_J_Nanotechnol-08-2116-s001.pdf]

Supporting Information

for

In situ controlled rapid growth of novel high activity TiB_2/(TiB_2–TiN) hierarchical/heterostructured nanocomposites

Jilin Wang[1], Hejie Liao[1], Yuchun Ji[1], Fei Long[1,*], Yunle Gu[2], Zhengguang Zou[1], Weimin Wang*[3], and Zhengyi Fu[3]

Address: [1]School of Materials Science and Engineering, Key Laboratory of Nonferrous Materials and New Processing Technology of Ministry of Education, Guilin University of Technology, Guilin 541004, China, [2]Nano and Ceramic Materials Research Center, Wuhan Institute of Technology, Wuhan 430073, China, and [3]The State Key Laboratory of Advanced Technology for Materials Synthesis and Processing, Wuhan University of Technology, Wuhan 430070, China

* Corresponding author

Email: Fei Long* - longf@glut.edu.cn; Weimin Wang* - shswmwang@whut.edu.cn

Additional TG-DSC results

Table 1: The major related TG-DSC results and the average particle sizes of the samples.

**Sample**	**Particle size**	**TG Temperature range**	**DSC exothermic peak temperature**	**DSC exothermic peak area**	**TG weight change**
#1-Commercial pure TiB_2 (contrast sample)	5um	1-463℃~550℃	Peak 1-518℃	Peak 1< Peak 2	↑9%
		2-550℃~839℃	Peak 2-710℃		↑82%
#2-0% endothermic rate	0.9um	1-446℃~577℃	Peak 1-490℃	Peak 1≈ Peak 2	↑28%
		2-577℃~707℃	Peak 2-644℃		↑45%
#3-40% endothermic rate	0.15um	1-422℃~517℃	Peak 1-480℃	Peak 1> Peak 2	↑35%
		2-517℃~695℃	Peak 2-630℃		↑27%
